# The Effects of Boredom on EFL Learners' Engagement

**DOI:** 10.3389/fpsyg.2021.743313

**Published:** 2021-09-06

**Authors:** Jin Xie

**Affiliations:** School of College English Teaching and Research, Henan University, Kaifeng, China

**Keywords:** boredom, engagement, learning, teaching, strategies

## Abstract

This article aims to delve into the role of boredom on students' engagement which has always attracted attention in that it is one of the most common academic feelings felt by students that causes them to feel more or less enthusiastic to engage in class activities, and there is a growing increase in such feelings among adolescents; therefore, its various dimensions should be taken into consideration. First and foremost, a variety of definitions from different points of view have been proposed. Then, with a focus on the distinction between state boredom and trait boredom which is one of the most radical classifications of boredom it has been continued. Following that, the antecedents of boredom are additionally taken into consideration along with the role of boredom in students' engagement that necessitates a few changes in the curriculum of schools. Moreover, some coping strategies on how to overcome boredom have been featured. Finally, in the discussion part, the emphasis of the points, which have been mentioned above, in the learning context for both teachers and students has been discussed, and new suggestions for further studies have been proposed.

## Introduction

### Boredom in Literature

In Charles Baudelaire's (2012) (as cited in Pawlak et al., 2020a, p. 3) interpretation, boredom is viewed as a complex problem that should be struggled with to create creativity. In other words, it is associated with an internal element of creativity. In his writing, boredom has been perceived as an action-provoking force that urges people to seek something through which they can feel alive. Additionally, boredom has been featured in Joseph Brodsky's (1995) (as cited in Pawlak et al., 2020a, p. 3) essays as a psychological Sahara characterized by the infinity of time, tedium, duplication, increasing sense of belittlement, diminishing any signs of self-praise. It is also mentioned that boredom should be embraced so that its mechanisms can be perceived (Pawlak et al., 2020a).

### Definition of Boredom

Not surprisingly, there is no fixed accepted definition for boredom because it is a complex construct that is rooted in a variety of factors and many other factors are impacted by it (Caldwell et al., 1999; Ally, 2008). The following feelings are accompanied by boredom: tedium, anguish, listlessness, lethargy, doldrums, or languor. The highly disturbing nature of boredom is also revealed by the aforementioned factors (Goldberg et al., 2011). Boredom in psychology can be regarded as a permanent (trait, it is a personality attribute) and temporary (state, it arises due to the situation) affective experience by which the learning process is severely inhibited and lack of interest in the class activities occur (Daniels et al., 2015). Lack of interest includes when the desire for partaking in the class activities is lowered and learners are inclined to escape such a context. Boredom results in feeling disengaged, therefore leading to avoidance behaviors. Consequently, there is a cause-and-effect link between lack of interest and boredom in which the former may lead to the latter (Pekrun et al., 2011).

From a societal aspect, boredom is viewed as a resistance to school rules and limitations forced by the educational system. Boredom defined by Barbalet (1999) is a high arousal state through which one can feels a sense of restlessness and irritability. However, according to Harris (2000), a sense of low arousal and dissatisfaction when feeling bored ascribes to an environment that is not sufficiently stimulating. As has been shown above, boredom can be defined from different aspects, which *per se* makes it a complex, dynamic, and multifaceted phenomenon.

### State Boredom vs. Trait Boredom

Boredom can be viewed as a more situation-based feature (Pawlak et al., 2020b). State boredom is referred to as a temporary, context-based, short-term condition that is rooted in an individual's understanding of their environment of learning which is not stimulating enough and it is impoverished (Bench and Lench, 2013). As for trait boredom, it is an inherent part of one's personality. This type of boredom is categorized into two classifications, external boredom proneness which is relevant to an uninteresting environment, and internal boredom proneness that refers to an individual having difficulty in involving things to do (Macklem, 2015).

### The Antecedents of Boredom

Many models have been put forward considering the causes of boredom. Out of which some are mentioned here. A method formulated, known as the attentional theory of boredom proneness (Harris, 2000; LePera, 2011) in which boredom is experienced as a result of the students' expectations and interests which have not been met and that is the reason why time seems to slow down for them and self-sustained attention has to be generated by students rather attentional control.

The control-value theory of achievement emotions (Pekrun, 2006; Tulis and Fulmer, 2013; Li, 2021) asserts if no value is found through the completion of a task, then the students are obliged to implement it whether they like it or not, hence, they are more likely to see boredom emerging with its avoidance behaviors. To put it simply, students feeling more valued and competent are less likely to feel bored. The Menton Theory of Boredom (Davies and Fortney, 2012) in which mentons referred to the usage of mental energy units known as mentons. It stipulates that when individuals receive exposure to over-challenging or under-challenging tasks, they feel bored. Surprisingly the fruit of being motivated to seek new incentives so as to do something with tasks can occur as a consequence of boredom.

According to a study carried out, the following factors contributing to an imbalance between what a learner feel inside and the external classroom factors: when a learner's comprehension is lowered, not having enough L2 skills, the difficulty level of tasks, when learners are bombarded with a lot of input, and not coming up with new ideas. All the aforementioned points have been found as the reasons behind boredom (Nakamura et al., 2021).

Likewise, four classifications have been formulated for the causes of boredom in online classes: teacher-related factors such as instructional practices and personality, IT/computer-related factors such as nature of online classes and computer literacy, task-related factors, such as task overload, task difficulty, and dull materials and subjects, and student-related factors, such as unmotivated students and students' irrelevant talk (Derakhshan et al., 2021). Similarly, based on a study conducted, boredom was experienced from low to moderate level by the learners attending online classes rather than traditional face-to-face classes; thus, a new type of boredom that can be found in language online classes should be analyzed and handled (Li and Dewaele, 2020).

### Disengagement and Boredom

One of the components which are adversely affected by boredom is engagement leading to a lower level of performance. To prevent disengagement, boredom should be overcome (Macklem, 2015). Engagement is an indispensable part of the process of learning and a multifold phenomenon. It has been classified into different categories: Behavioral engagement such as the effort; emotional engagement such as high levels of enthusiasm which is associated with low levels of anxiety and boredom; cognitive engagement such as the usage of learning strategy and self-regulation; agentic engagement such as the amount of conscious effort so that the learning experience would be enriched (Veiga et al., 2014; Hiver et al., 2021). Out of the afore-mentioned categories, the one which is highly important in the learning process is behavioral engagement in that it is pertinent to the actual recognition of an individual's learning talents (Dörnyei, 2019; Oga-Baldwin, 2019).

Another possibility that can be viewed is to consider engagement from two other aspects, internal and external. The former implies that how much time and effort is allocated to the process of the learning while the latter entails the measures that are taken at the institutional level so that the resources would be dealt with along with other options of learning and services for support which encourage the involvement in activities resulting in the likely outcomes such as consistency and satisfaction (Harper and Quay, 2009; Kuh, 2009). Much attention is deserved to be paid to engagement since it is perceived as a behavioral means with which students' motivation can be realized and as a consequence, development through the process of learning can happen (Jang et al., 2010). Active involvement should be strengthened in L2 classes to prevent disruptive behaviors and diminish the valence of emotions which are negative such as feeling anxious, frustrated, and bored.

It has been claimed by some writers (e.g., Skinner, 2016) disengagement itself does not happen frequently in educational settings due largely to the fact that it is relevant to extreme behaviors, and it is when another phrase disaffection can be considered significant. Disaffection is characterized by disinterest, aversion, resignation, and reduced effort. Therefore, our perception of boredom as a complex emotion can be enhanced, and it can be dealt with more systematically if boredom is viewed through the following factors, disengagement and disaffection. In language learning context and use, engagement with learning a language is a cognitive, affective, and/or social process where the learner is viewed as the agent and language is regarded as the object. The learner is engaged:

• “Cognitively: the engaged individual is alert, complete attention is focused, and their own knowledge is constructed.

• Affectively: the engaged individual has a positive, intentional, willing, and independent approach toward the language and/or what it represents.

• Socially: the engaged individual is interactive and initiating” (Svalberg, 2009, p. 247).

### Coping Strategies About Boredom in Educational Settings

Students should be trained to accept boredom as a transient lack of stimulation and to understand their needs and feelings to attend to the tasks with more diligence, sobriety, and engagement (Eastwood et al., 2007). Boredom can be reduced by providing students with options so that they could feel more autonomous in the process of learning. To lower boredom, mastery goals through which the real meaning of perseverance can be shown can additionally be enhanced. It enables students to be less susceptible to surrender in negative situations and causes them to have more determination to find task-based self-improving activities (Pekrun, 2006; Turner and Husman, 2008; Furner and Gonzalez-DeHass, 2011).

Last but not least, teachers' enthusiasm about their academic value and about the content of the classes in addition to respecting students' requirements and attitudes is another factor that helps one minimize the possibility of feeling bored. Another factor leading to a reduction in boredom is that giving students positive affective feedback which leads to believing in their own abilities and overcoming boredom. It has also been reported that even in online classes students have an inclination to be engaged in discussions and it is what makes the class more interactive and challenging (Derakhshan et al., 2021).

## Conclusion

In this article several parts have been discussed, the definition of boredom, the causes of boredom, the effect of boredom on students' engagement, and how boredom can be coped with. It should be taken into account that teachers are not just responsible for conveying the pedagogical knowledge in classes, instead what seems essentially vital is the amount to which teachers make an effort to make the class more interactive and make the tasks more challenging so that students would feel more focused and less bored. It turned out engagement is the key to not feeling bored. The more engaged the students are, the less bored they feel and the less monotonous they find the class activities. Without a shadow of a doubt, further studies can be conducted to aid students not to feel bored and to actively participate in the class activities. Thanks to cutting-edge technology, everything has been revolutionized; therefore, new types of boredom that are pertinent to online learning environments, for example short attention span and students' low literacy of computers, had better be carried out (Kruk, 2021; Wang and Derakhshan, 2021).

## Author Contributions

JX independently drafted the manuscript and revised it with great care before it was submitted to this special issue.

## Conflict of Interest

The author declares that the research was conducted in the absence of any commercial or financial relationships that could be construed as a potential conflict of interest.

## Publisher's Note

All claims expressed in this article are solely those of the authors and do not necessarily represent those of their affiliated organizations, or those of the publisher, the editors and the reviewers. Any product that may be evaluated in this article, or claim that may be made by its manufacturer, is not guaranteed or endorsed by the publisher.
